# The Influence of Heavy Metals on Gastric Tumorigenesis

**DOI:** 10.1155/2022/6425133

**Published:** 2022-05-28

**Authors:** Liang Wang, Congxiu Miao, Yuan He, Hanglong Li, Shasha Zhang, Keyan Li, Huimin Liu, Wushuang Li, Jiangman Zhao, Yue Xu, Hui Tang, Qiang Zhao

**Affiliations:** ^1^Department of Gastrointestinal Surgery, Heping Hospital, Changzhi Medical College, Changzhi 046000, China; ^2^Department of Reproductive Genetics, Heping Hospital, Changzhi Medical College, Key Labrotory of Reproduction Engineer of Shanxi Health Committee, Changzhi 046000, China; ^3^Shanghai Biotecan Pharmaceuticals Co., Ltd., 180 Zhangheng Road, Shanghai 201204, China; ^4^Shanghai Zhangjiang Institute of Medical Innovation, Shanghai 201204, China

## Abstract

**Objectives:**

This study aimed to observe the relationship among heavy metals concentration, microsatellite instability (MSI), and human epidermal growth factor receptor type 2 (HER2) gene amplification in gastric cancer (GC) patients.

**Methods:**

The concentrations of 18 heavy metals in the plasma of GC patients and healthy controls were measured by inductive coupled plasma emission spectrometry (ICP-MS). MSI detection was conducted by detecting 5 microsatellite repeat markers by PCR analysis. HER2 gene amplification was detected by fluorescence in situ hybridization (FISH). The relationship among heavy metal elements, tumor biomarkers, HER2 amplification, and MSI status was analyzed by Pearson correlation analysis.

**Results:**

A total of 105 GC patients and 62 healthy controls were recruited in this study. The concentration of arsenic (As), chromium (Cr), cuprum (Cu), mercury (Hg), manganese (Mn), lead (Pb), stibium (Sb), selenium (Se), stannum (Sn), strontium (Sr), thallium (Tl), vanadium (V), and zinc (Zn) were significantly different between GC patients and controls. Among 105 GC patients, including 87 microsatellite-stable (MSS) samples and 18 MSI samples, the concentration of Ga is significantly higher in the MSI group than that in the MSS group. Meanwhile, in 97 GC patients having detected HER2 gene amplification, 69 of 97 had negative HER2 gene amplification and the rest 28 GC patients had positive HER2 gene amplification. The concentration of Hg, Sn, and Tl is noticeably higher in the HER2 positive group than in the HER2 negative group. Only Sb was positively correlated with MSI, but none of these heavy metals was correlated with HER2 gene amplification.

**Conclusions:**

The results indicated that Sb has significant positive correlation with the MSI status, which suggests that Sb may cause MSI in GC. However, further research studies are required to elucidate the mechanisms in the near feature.

## 1. Introduction

Gastric cancer (GC) is the fifth most common cancer and the third leading cause of cancer mortality worldwide. About 1 million new GC patients are diagnosed and more than 783,000 patients die because of GC each year [1].

Studies have shown that the 5-year survival rate of early GC surgery could reach more than 90%, while the 5-year survival rate of postoperative GC is only 5%–15% [2]. Therefore, it is worthwhile to improve early GC diagnostic methods. However, most early GC patients have no obvious symptom, or just the symptoms of nausea, vomiting, or similar upper gastrointestinal tract [3]. In a previous study, CYFRA 21-1, a fragment of cytokeratin 19, was used as a reliable tumor marker for early GC [4]. Moreover, CYFRA 21-1 has been introduced as a potential marker for monitoring various types of diseases, including lung cancer [5]. In addition, many studies have shown that serum carbohydrate antigen 72-4 (CA72-4), carcinoembryonic antigen (CEA), and carbohydrate antigen 19-9 (CA19-9) can be used as biomarkers for early GC diagnosis [3, 6–8]. Studies have shown that CEA is the most widely investigated tumor biomarker in various tumors such as colorectal, pancreas, liver, breast, and lung tumors [9, 10]. Furthermore, Cha et al. showed that a low serum pepsinogen (sPG) I level and a low sPG I/II ratio level may serve as a sensitive biomarker for early GC patients [11]. However, their sensitivity and specificity are limited, and the diagnosis rate of early GC is relatively low. Therefore, most GC patients are diagnosed at middle or late stages. Even if they accept surgery, chemotherapy, and radiotherapy method, they may still have a poor outcome [12].

In recent years, although the incidence and mortality of GC are decreasing, both the detection rate and prognosis are not satisfactory [13]. The median overall survival (OS) after chemotherapy was 7.5–12.0 months and 3.0–5.0 months for the supportive care [14–16]. Therefore, it is urgent to find new therapeutic approaches in GC. Molecular targeted therapies could be promising methods. For example, anti-human epidermal growth factor receptor 2 (HER2) are the most widely used antibodies with clear clinical significance [17, 18].

HER2 (also known as ERBB2) gene is located on chromosome 17q2l and encodes a receptor transmembrane tyrosine kinase ERBB2, which is a member of the epidermal growth factor receptor (EGFR) family of receptor tyrosine kinases [19]. The proportion of HER2 overexpression/amplification in GC is 6.1%–23.0% [20, 21]. Overexpressed HER2 protein has been proved to be associated with the degree of tumor differentiation, tumor size, lymph node metastasis, and poor survival [22]. However, HER2 gene amplification is not always associated with its protein overexpression [23, 24]. Many studies demonstrated that targeted HER2 overexpression and/or amplification has been a prospective therapy in many cancers, including GC [25]. Several studies have confirmed the efficacy of trastuzumab for HER2-positive metastatic GC [26].

In addition, with the development of next-generation sequencing, microsatellite instability (MSI) has become an essential testing for applying immunotherapy and chemotherapy in patients with potentially resectable disease [27]. MSI commonly resulted from the functional deficiency of one or more mismatch repair (MMR) proteins [28]. The MSI status plays a pivotal role in the development of GC [29]. Moreover, microsatellite-instability-high (MSI-H) phenotype could serve as a predictive factor in cancer immunotherapy. Clinical trials evidence has demonstrated that patients with MSI phenotype had more responses to anti-PD1 monoclonal antibodies when compared with microsatellite-stable (MSS) patients who failed the traditional therapy [30–32]. On the contrary, GC patients with MSS status could benefit from 5-FU-based adjuvant chemotherapy in TNM stage II-III [33].

However, GC is a complex process and is affected by genetic and environmental factors. Several factors have been noted to have a significant impact on increasing the risk of developing GC, like age, sex, family history, diet, alcohol consumption, smoking, and *Helicobacter pylori* infection [27, 34]. In recent years, heavy metal exposure has been indicated as one of the environmental risk factors [35]. A previous study has revealed that heavy metal exposure is associated with the incidence and death of GC [36]. For example, heavy metals like chromium (Cr), nickel (Ni), and cadmium (Cd) may destroy the gastric mucosa barrier, causing inflammation and tissue damage, thus leading to GC. Many studies have been shown that heavy metals can induce reactive oxygen species (ROS), which causes DNA lesions and gastric mucosal damage, further leading to GC [37, 38]. However, currently there is no comprehensive description of the role of heavy metals exposure in the progression of GC, and the relationship among heavy metals, HER2 gene amplification, and MSI status remains unknown.

In this study, we recruited 105 GC patients and measured the concentration of 18 heavy metals, MSI status, and HER2 gene amplification through the methods of inductive coupled plasma emission spectrometry (ICP-MS), PCR, and fluorescence in situ hybridization (FISH), respectively. We aim to explore the correlation of heavy metals, MSI status, and HER2 gene amplification in GC and to reveal whether metals can influence MSI phenotype or HER2 gene amplification in GC.

## 2. Materials and Methods

### 2.1. Patients and Samples

One hundred five pathologically confirmed gastric cancer (GC) patients and 62 healthy control samples were recruited at Heping Hospital Affiliated to Changzhi Medical College from March 2020 to December 2020. All patients did not receive any therapies before surgery, including chemotherapy and radiotherapy. After surgery, 10% formalin fixed paraffin-embedded (FFPE) tumor and adjacent-tumor tissues were obtained to examine MSI and HER2 gene amplification.

All procedures were performed and approved (approval no. RT2020029) in accordance with the ethical standards of the Clinical Research Ethics Committee of Heping Hospital, and written informed consent was obtained from all participants.

### 2.2. MSI Detection

MSI detection is conducted by PCR amplification of specific microsatellite repeats, whose instability is determined by comparing the length of nucleotide repeats in tumor and adjacent tumor tissues. These regions are amplified from both tumor and nontumor tissues by fluorescent multiplex PCR, and their sizes were assessed by capillary electrophoresis [39, 40]. MSI detection was performed by a MSI panel including BAT25, BAT26, D2S123, D5S346, and D17S250 as described in [41]. In brief, DNA extraction was performed using a FFPE whole genome extraction kit (Qiagen, Hilden, Germany). PCR was assessed using the following process: 95°C for 2 min, 40 cycles of 95°C for 30 s, 55°C for 30 s, 72°C for 30 s and 7 min, and then maintaining at 4°C. After amplification, PCR products were detected and analyzed by capillary electrophoresis with a ABI 3730XL DNA analyzer (ABI, USA). Microsatellite-instability-low (MSI-L) was defined as one MSI marker showing instability, and MSI-H was defined as at least 2 of 5 MSI markers showing instability, while MSS was defined as no instability in tumor DNA as previously described [41].

### 2.3. FISH Assay

HER2 gene amplification was assessed by FISH, which is regarded as the gold standard as described [42, 43]. FISH was carried out on FFPE tumor tissue sections using the Fast Probe (FP-001) kit from Wuhan HealthCare Biotechnology Co., Ltd. (Wuhan, China) according to the manufacturer's instructions. The probes for detecting HER2 gene status are double probes containing the HER2 gene and the centromeric region of chromosome 17 (CEP17) that is used to label the HER2 gene location [44]. More than 75% of the tumor cell nuclei had a hybridization signal, and at least 30 nonoverlapping cancer cells with complete boundaries were counted using 100× objective lens. The ratios of HER2/CEP17 were calculated as follows: the HER2/CEP17 ratio ≥2.0 and average HER2 copies/cells ≥4.0 were defined as HER2 gene positive amplification, while the HER2/CEP17 ratio <2.0 and average HER2 copies/cells<4.0 were defined as HER2 gene negative amplification, as described in [45].

### 2.4. ICP-MS Detection

About 2 ml of whole blood were collected from each sample. Serum was separated by centrifugation at 3000 rpm/10 min, and then was stored at −20°C until further analysis. The level of 18 heavy metals, including vanadium (V), chromium (Cr), manganese (Mn), cobalt (Co), nickel (Ni), cuprum (Cu), zinc (Zn), gallium (Ga), arsenic (As), selenium (Se), strontium (Sr), cadmium (Cd), stannum (Sn), stibium (Sb), barium (Ba), mercury (Hg), thallium (Tl), and lead (Pb), were obtained by ICP-MS (Agilent 7800), a well-used technique for multielemental capabilities analysis, according to the manufacturer's instructions as described in [46].

### 2.5. Traditional Serum Biomarker Detection

10 mL of venous blood was obtained from each participant, blood samples were centrifuged at 3000 rpm for 15 mins at 4°C for 1 h, and the upper serum was collected. The standard solutions of CA19-9, CEA, and CA72-4 were prepared in buffer solution (pH = 7.5) using the serial dilution method as described [8]. Then, serum tumor markers CA72-4, CA19-9, and CEA were detected by chemiluminescence immunoassay as described [3].

### 2.6. Statistical Analysis

Categorical variables were presented by numbers, and differences in distribution between the two groups were analyzed using the chi-squared test in SPSS 19.0 software. Continuous variables are presented as the median with interquartile range (*IQR)*. Continuous variables between the two groups were compared using the Wilcoxon rank-sum test (also known as the Mann–Whitney *U*-test) in SPSS 19.0 or R studio (v.3.6.1). Correlations among 18 heavy metals, MSI status, HER2 gene amplification, and 3 traditional biomarkers were analyzed by Pearson correlation analysis using R studio. *p* < 0.05 was considered statistically significant.

## 3. Results

### 3.1. Characteristics of the GC Patients

According to the results of MSI detection, 87 samples were the MSS phenotype (the MSS group) and other 18 samples were the MSI phenotype (the MSI group). The characteristics of these two groups are shown in Table 1. There was no significant difference between the MSS and MSI groups in terms of age, sex, BMI, diabetes mellitus, history of CAD, family tumor history, smoking/alcohol consumption status, tumor size, tumor stage, the degree of differentiation, and HER2 gene amplification. However, hypertension (*p*=0.034), perineural invasion (*p*=0.022), lymphovascular invasion (*p*=0.004), lymph node metastasis (*p*=0.009), and distant metastasis(*p*=0.042) were significantly different between the two groups.

### 3.2. Comparison of Metal Concentrations in Gastric Patients and Healthy Controls

**Figure 1:**
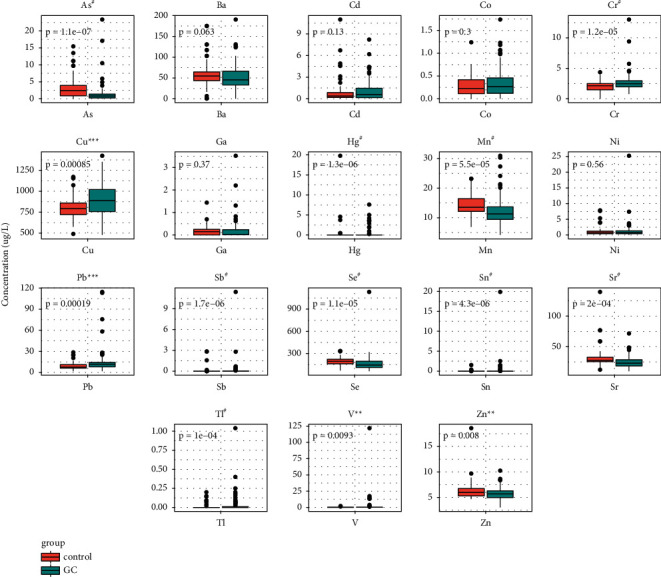
Comparison of 18 heavy metals between healthy controls and GC patients. Statistical analysis was performed by the Wilcoxon rank-sum test. ^*∗*^*p* < 0.05, ^*∗∗*^*p* < 0.01, ^*∗∗∗*^*p* < 0.0001, and ^#^*p* < 0.00001.

In order to compare the 18 heavy metals in the serum between healthy controls and GC patients, we recruited 62 healthy control samples. There was significant difference of heavy metal concentrations between the two groups, except for Ba, Cd, Co, Ga, and Ni (Figure 1, data shown in Table S1).

The median concentration with IQR for each heavy metals in the control group and the GC group are as follows: Cr 2.15 (1.45–2.51) *μ*g/L vs. 2.47 (1.95–2.99) *μ*g/L, Cu 792.30 (720.60–860.10) *μ*g/L vs. 886.80 (755.50–1024) *μ*g/L, Hg 0(0–0) *μ*g/L vs. 0 (0–0.01) *μ*g/L, Pb 7.42 (5.97–11.71) *μ*g/L vs. 11.76 (7.97–14.41) *μ*g/L, Sb 0(0–0) *μ*g/L vs. 0.01 (0–0.03) *μ*g/L, Sn 0 (0–0) *μ*g/L vs. 0.01 (0.00–0.01) *μ*g/L, Tl 0 (0–0) *μ*g/L vs. 0 (0–0.01) *μ*g/L, and V 0.17 (0.05–0.44) *μ*g/L vs. 0.26 (0.16–0.50) *μ*g/L. The results indicated that Cr, Cu, Hg, Pb, Sb, Sn, Tl, and V were significantly higher in the GC group (Figure 1).

However, the median concentration with IQR for each heavy metals in the control group and the GC group are as follows: As 2.44 (0.84–4.08) *μ*g/L vs. 0.82 (0.22–1.44) *μ*g/L, Mn 13.55 (12.05–16.49) *μ*g/L vs. 11.28 (9.42–13.87) *μ*g/L, Se 193.60 (158.20–224.80) *μ*g/L vs. 143.90 (110.30–196.30) *μ*g/L, Sr 27.70 (25.24–33.27) *μ*g/L vs. 23.06 (18.33–29.07) *μ*g/L, and Zn 5.98 (5.29–6.76) *μ*g/L vs. 5.68 (4.92–6.32) *μ*g/L. The results indicated that As, Mn, Se, Sr, and Zn were significantly lower in the GC group (Figure 1).

### 3.3. Comparison of Metal Concentrations and Traditional Biomarkers in the MSS and MSI Groups

**Figure 2:**
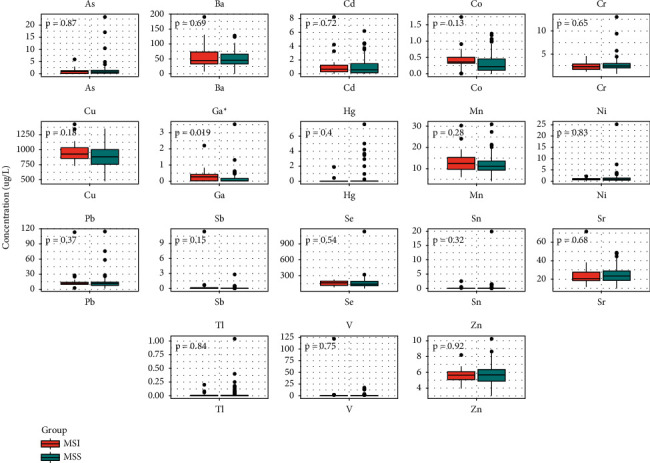
Comparison of 18 heavy metals between the MSS group and the MSI group. Statistical analysis was performed by the Wilcoxon rank-sum test. ^*∗*^*p* < 0.05.

We also compared 18 heavy metals between the MSS and MSI groups. However, only the median concentration with IQR of Ga was noticeably lower in the MSS group than that in the MSI group, which is 0.01 (0.01–0.19) *μ*g/L vs. 0.26 (0.01–0.46) *μ*g/L (Figure 2, data shown in Table S2). In addition, 48 of 105 GC patients had been tested for CEA, CA19-9, and CA72-4, and we compared these three biomarkers between the MSS (38 cases) and MSI (10 cases) groups. The results indicated that only the median level with IQR of CA72-4 was significantly higher in the MSI group than that in the MSS group, which is 4.03 (2.07–19.54) U/mL vs. 1.64 (1.05–4.94) U/mL (Figure S1, data shown in Table S3).

### 3.4. Comparison of Metal Concentrations and 3 Biomarkers in the HER2 Negative and Positive Expression Groups

**Figure 3:**
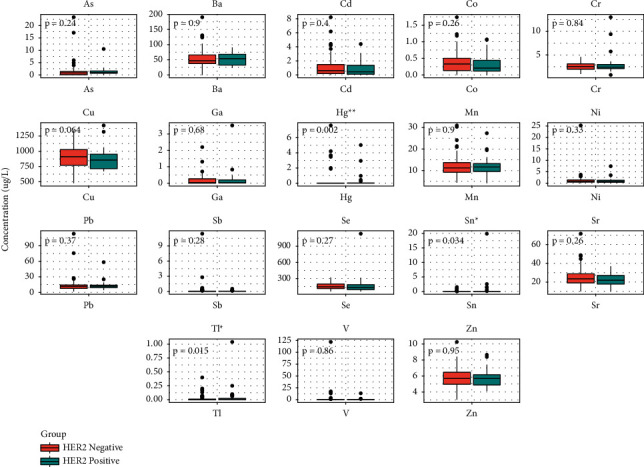
Comparison of 18 heavy metals between the HER2 negative group and the HER2 positive group. Statistical analysis was performed by the Wilcoxon rank-sum test. ^*∗*^*p* < 0.05 and ^*∗∗*^*p* < 0.01.

In 97 GC patients who have been tested for the HER2 gene amplification, it is observed that 69 of 97 patients had HER2 negative expression and 28 of 97 had HER2 positive expression. The median concentration (with IQR) of Hg, Sn, and Tl were significantly higher in the HER2 positive group than those in the HER2 negative group, which is 0.01 (0–0.01) *μ*g/L vs. 0 (0–0.01) *μ*g/L, 0.01 (0–0.01) *μ*g/L) vs. (0 (0–0.01) *μ*g/L, and 0.01 (0–0.05) *μ*g/L vs. 0 (0–0.01) *μ*g/L, respectively (Figure 3, data shown in Table S4). Besides that, among 97 samples, 44 cases have been tested for CEA, CA19-9, and CA72-4, and the three biomarkers were compared between the HER2 negative (31 cases) and positive (13 cases) groups. However, no significant difference expression was observed between the two groups (Figure S2, data shown in Table S5).

### 3.5. Correlations Analysis among MSI, HER2 Amplification, and Heavy Metals

**Figure 4:**
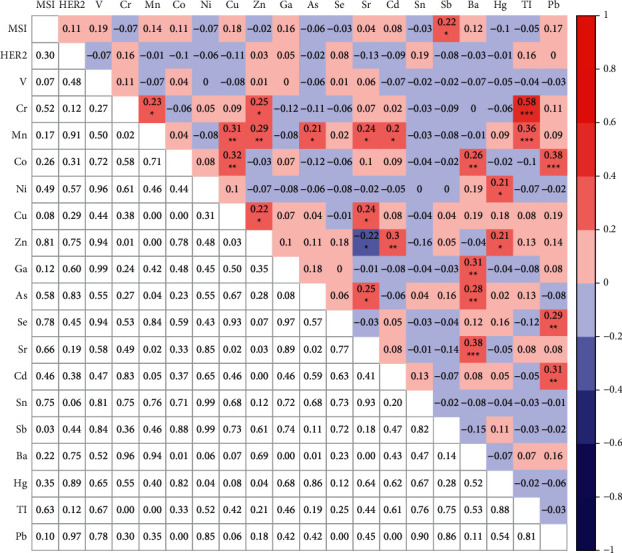
Correlations analysis among MSI, HER2 gene amplification, and 18 heavy metals. ^*∗*^*p* < 0.05, ^*∗∗*^*p* < 0.01, and ^*∗∗∗*^*p* < 0.0001.

In 97 GC patients who have been tested for the MSI status, HER2 gene amplification, and 18 heavy metals, correlations among these three clinical characteristics were analyzed. No significant correlations were found between the HER2 gene amplification and MSI status, nor between HER2 amplification and any of these metals. Only the concentration of Sb was significantly positively correlated with the MSI status (*r* = 0.22, *p* < 0.05) (Figure 4, data shown in Table S6). Moreover, correlation among these metals were analyzed. Significant associations were found between most of these metals. Specifically, Cr, Mn, Co, Ni, Cu, Zn, Ga, As, Se, Sr, and Cd showed significant correlations with other metals. Surprisingly, Zn and Sr showed negative correlation with each other (*r* = −0.22, *p* < 0.05) (Figure 4).

**Figure 5:**
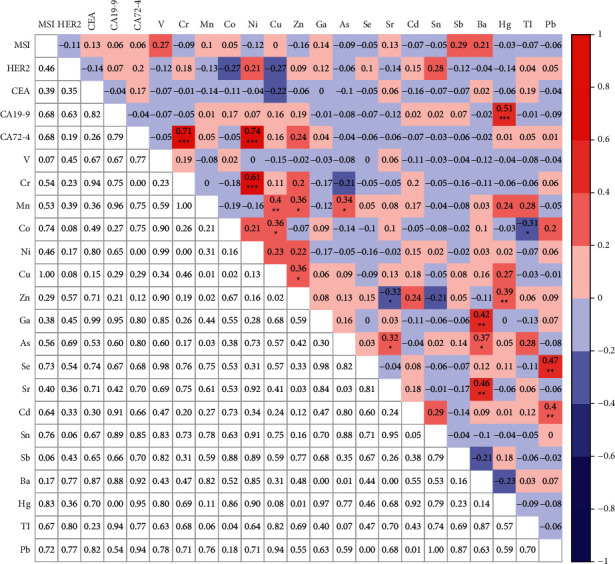
Correlations analysis among MSI, HER2 gene amplification, 3 biomarkers, and 18 heavy metals. ^*∗*^*p* < 0.05, ^*∗∗*^*p* < 0.01, and ^*∗∗∗*^*p* < 0.0001.

In addition, 44 of 97 patients also showed the 3 traditional biomarkers. Thus, we analyzed correlation among all the clinical characteristics tested in these patients. No significant correlation was found among the MSI status, HER2 gene amplification, and 18 heavy metals. Surprisingly, CA19-9 was significantly positively correlated with Hg (*r* = 0.51, *p* < 0.001). CA72-4 was strongly positively correlated with Cr (*r* = 0.71, *p* < 0.001) and Ni (*r* = 0.74, *p* < 0.001). Moreover, Cr, Mn, Co, Cu, Zn, Ga, As, Se, Sr, and Cd showed significant correlations with other heavy metals. Interestingly, significant negative correlation was found between Zn and Sr (*r* = −0.32, *p* < 0.05) and between Co and Tl (*r* = −0.31, *p* < 0.05) (Figure 5, data shown in Table S7).

## 4. Discussion

In the present study, 105 GC patients have been tested for the MSI status and 87 patients were MSS and 18 patients were MSI. We collected 16 clinical characteristics and analyzed their associations with the MSI status. The results indicated that the hypertension, perineural invasion, lymphovascular invasion, lymph node metastasis, and distant metastasis were significantly associated with the MSI status in GC.

In addition, we compared the 18 heavy metals between 62 healthy controls and 105 GC patients. The results indicated that the median concentrations of Cr, Cu, Hg, Pb, Sb, Sn, Tl, and V were significantly lower in the healthy controls than that in GC patients. Bibi et al. reported that the concentration of Pb and Cr were found to be significantly higher in thyroid cancer patients than in healthy controls [47]. On the contrary, the median of As, Mn, Se, Sr, and Zn were noticeably higher in the control group than in the GC group. Cd, Pb, and Hg can disrupt endocrine activities and restrict DNA reconstructions. Much evidence reported that Se, Zn, Cd, Cr, and As are involved in several various biological mechanisms including cell stabilization, DNA damage response and repair, and tumor progression [48–50]. Moreover, MLH1, one of the important MMR proteins, is regulated in activation of the G2/M cell cycle checkpoint in response to Cr exposure [51]. Processing of Cr-induced DNA damage by MMR causes the extensive formation of *γ*-H2AX foci in the *G*_2_ phase [52]. In addition, Jin et al. utilized a yeast model system to demonstrate that low concentrations of Cd inhibit MMR in yeast cells and in *in vitro* cell-free extracts [53]. Oliveira et al. used a panel of six microsatellite loci to detect the genotoxic effects of Cd in murine testes. They detected MSI in two of the five tested microsatellite markers in Cd-exposed mouse testes [54].

Besides that, the concentration of 18 heavy metals were compared between 18 MSI patients and 87 MSS patients. The concentration of Ga was significantly higher in the MSI group than that in the MSS group. Hg, Sn, and Tl were significantly higher in the HER2 positive group than that in the HER2 negative group. However, there is no research about these findings yet. Therefore, heavy metals may contribute to the MSI status and HER2 gene expression, but the pathogenic mechanism needs to be further studied. Besides that, the level of serum tumor biomarkers plays an important role in the progression of GC [55]. A previous study indicated that CA72-4 was correlated with the TNM stage in GC patients [56]. In this study, we found that CA72-4 was significantly higher in the MSI group than in the MSS group. However, no significant difference biomarker expression between the HER2 negative and positive groups.

Last but not least, we analyzed the correlations among the MSI status, HER2 gene amplification, and 18 heavy metals in 97 samples. We only found that Sb was noticeably positively correlated with the MSI status (r = 0.22, *p* < 0.05). However, no research reported the correlation of heavy metals, MSI status, and HER2 gene amplification yet. Thus, large samples are needed to verify the results in the near feature. Moreover, Cr, Mn, Co, Ni, Cu, Zn, Ga, As, Se, Sr, and Cd showed significant correlations with other heavy metals. Feng et al. have showed that Cr and Ni showed strong correlations with other metals in GC [57]. In addition, we also analyzed correlation among 3 traditional biomarkers, 18 heavy metals, MSI status, and HER2 gene expression in 44 GC patients. Interestingly, we found that CA19-9 was significantly positively correlated with Hg, and CA72-4 was strongly positively correlated with Cr and Ni.

Still, there are several limitations in this study. First, the sample size was relatively small and unbalanced for many groups. Second, as for biomarkers detection, only 48 of 105 samples detected CEA, CA19-9, and CA72-4; thus the results need to be verified by larger sample sizes in the near feature.

## 5. Conclusions

In the present study, we found that the concentrations of 13 of 18 heavy metals have significant difference between healthy controls and GC patients. Ga was significantly higher in the MSI group than that in the MSS group. Furthermore, Hg, Sn, and Tl were significantly higher in the HER2 positive group than that in the HER2 negative group. Importantly, Sb has significant positive correlation with the MSI status. Therefore, more research studies should be carried out to reveal these mechanisms in the near feature.

## Figures and Tables

**Table 1:** Clinical characteristics for 105 GC patients enrolled in this study.

Items	Total (105)	MSI (n=18)	MSS (n=87)	*P* value
Sex				0.434
Male	80	15	65	
Female	25	3	22	
Age (median with IQR)		59.50 (52.25–66.25)	64.00 (57.00–67.00)	0.304
BMI (kg/m^2^) (median with IQR)		22.18 (18.37–23.44)	22.65 (20.31–24.49)	0.299
Hypertension				0.034
Yes	34	2	32	
No	71	16	55	
Diabetes mellitus				0.154
Yes	9	0	9	
No	96	18	78	
CAD history				0.062
Yes	7	3	4	
No	98	15	83	
Family tumor history				0.075
Yes	4	2	2	
No	101	16	85	
Smoking				0.944
Yes	24	4	20	
No	81	14	67	
Alcohol consumption				0.354
Yes	4	0	4	
No	101	18	83	
Tumor size (cm) (median with IQR)		5.00 (3.00–7.00)	4.50 (3.00–6.00)	0.518
NA	5	1	4	
Tumor stage				0.073
I-II	48	9	39	
III-IV	56	8	48	
NA	1	1	0	
Degree of tumor differentiation				0.571
low	68	10	58	
middle	34	7	27	
high	3	1	2	
Perineural invasion				0.022
Yes	50	5	45	
No	54	12	42	
NA	1	1	0	
Lymphovascular invasion				0.004
Yes	47	3	44	
No	57	14	43	
NA	1	1	0	
Lymph node metastasis				0.009
Yes	72	8	64	
No	32	9	23	
NA	1	1	0	
Distant metastasis				0.042
Yes	10	3	7	
No	94	14	80	
NA	1	1	0	
HER2 gene amplification				0.230
Positive	28	7	21	
Negative	69	11	58	
NA	8	0	8	

GC: gastric cancer; NA, not application; IQR: interquartile range; BMI, body mass index; MSI, microsatellite instability; MSS, microsatellite stable; CAD, coronary artery disease; *P* < 0.05 was considered significant.

## Data Availability

The data used to support the findings of this study are included within the article.

## Abbreviations

MSI: Microsatellite instability
HER2: Human epidermal growth factor receptor type 2
GC: Gastric cancer
ICP-MS: Inductive coupled plasma emission spectrometry
FISH: Fluorescence in situ hybridization
MSS: Microsatellite-stable
MMR: Mismatch repair
CEP17: Centromeric region of chromosome 17
EGFR: Epidermal growth factor receptor
MSI-H: Microsatellite-instability-high
ROS: Reactive oxygen species
FFPE: Formalin fixed paraffin-embedded
MSI-L: Microsatellite-instability-low
V: Vanadium
Cr: Chromium
Mn: Manganese
Co: Cobalt
Ni: Nickel
Cu: Cuprum
Zn: Zinc
Ga: Gallium
As: Arsenic
Se: Selenium
Sr: Strontium
Cd: Cadmium
Sn: Stannum
Sb: Stibium
Ba: Barium
Hg: Mercury
Tl: Thallium
Pb: Lead
CEA: Carcinoembryonic antigen
CA19-9: Carbohydrate antigen 19-9
CA72-4: Carbohydrate antigen 72-4.

## Supplementary Materials

Supplementary Materials Figure S1: comparison of CEA, CA19-9, and CA72-4 between the MSS group and the MSI group. Statistical analysis was performed by the Wilcoxon rank-sum test. ^*∗*^*p* < 0.05. Figure S2: comparison of CEA, CA19-9, and CA72-4 between the HER2 negative group and the HER2 positive group. Table S1: comparison of 18 heavy metals between healthy controls and GC patients. Table S2: comparison of 18 heavy metals between the MSS group and the MSI group. Table S3: comparison of CEA, CA19-9, and CA72-4 between the MSS group and the MSI group. Table S4: comparison of 18 heavy metals between the HER2 negative group and the HER2 positive group. Table S5: comparison of CEA, CA19-9, and CA72-4 between the HER2 negative group and the HER2 positive group. Table S6: correlations analysis among MSI, HER2 gene amplification, and 18 heavy metals. Table S7: correlations analysis among MSI, HER2 gene amplification, 3 biomarkers, and 18 heavy metals.
