# Supplementary material for: The Influence of Heavy Metals on Gastric Tumorigenesis
Source: J Oncol. 2022 May 28;2022:6425133. doi: 10.1155/2022/6425133 (PMC9167133; doi:10.1155/2022/6425133)
Supplement: Supplementary Materials — Figure S1: comparison of CEA, CA19-9, and CA72-4 between the MSS group and the MSI group. Statistical analysis was performed by the Wilcoxon rank-sum test. ∗p < 0.05. Figure S2: comparison of CEA, CA19-9, and CA72-4 between the HER2 negative group and the HER2 positive group. Table S1: comparison of 18 heavy metals between healthy controls and GC patients. Table S2: comparison of 18 heavy metals between the MSS group and the MSI group. Table S3: comparison of CEA, CA19-9, and CA72-4 between the MSS group and the MSI group. Table S4: comparison of 18 heavy metals between the HER2 negative group and the HER2 positive group. Table S5: comparison of CEA, CA19-9, and CA72-4 between the HER2 negative group and the HER2 positive group. Table S6: correlations analysis among MSI, HER2 gene amplification, and 18 heavy metals. Table S7: correlations analysis among MSI, HER2 gene amplification, 3 biomarkers, and 18 heavy metals. [file 6425133.f1.zip › 6425133.f1/Table S1.docx]

| Table S1: Comparison of 18 heavy metals between the healthy controls and the GC patients. | | | |
| --- | --- | --- | --- |
|  | control (n=62) | GC (n=105) |  |
| Heavy metals | Median+IQR | Median+IQR | *p* value |
| V | 0.17 (0.05-0.44) | 0.26 (0.16-0.50) | 0.0093 |
| Cr | 2.15 (1.45-2.51) | 2.47 (1.95-2.99) | 1.2E-05 |
| Mn | 13.55 (12.05-16.49) | 11.28 (9.42-13.87) | 5.5E-05 |
| Co | 0.23 (0.11-0.42) | 0.27 (0.12-0.47) | 0.3 |
| Ni | 0.69 (0.41-1.24) | 0.77 (0.41-1.36) | 0.56 |
| Cu | 792.30 (720.60-860.10) | 886.80 (755.50-1024) | 0.00085 |
| Zn | 5.98 (5.29-6.76) | 5.68 (4.92-6.32) | 0.008 |
| Ga | 0.16 (0-0.26) | 0.01 (0.01-0.25) | 0.37 |
| As | 2.44 (0.84-4.08) | 0.82 (0.22-1.44) | 1.1E-07 |
| Se | 193.60 (158.20-224.80) | 143.90 (110.30-196.30) | 1.1E-05 |
| Sr | 27.70 (25.24-33.27) | 23.06 (18.33-29.07) | 2.0E-04 |
| Cd | 0.38 (0.16-0.92) | 0.6 (0.16-1.48) | 0.13 |
| Sn | 0 (0-0) | 0.01(0-0.01) | 4.3E-06 |
| Sb | 0 (0-0) | 0.01(0-0.03) | 1.7E-06 |
| Ba | 54.55 (42.86-65.78) | 45.33 (32.77-66.69) | 0.063 |
| Hg | 0 (0-0) | 0 (0-0.01) | 1.3E-06 |
| Tl | 0 (0-0) | 0 (0-0.01) | 1.0E-04 |
| Pb | 7.42 (5.97-11.71) | 11.76 (7.97-14.41) | 0.00019 |
| GC: gastric cancer; IQR: interquartile range. | | | |

**p*<0.05 was considered significant.
