# Supplementary material for: The Influence of Heavy Metals on Gastric Tumorigenesis
Source: J Oncol. 2022 May 28;2022:6425133. doi: 10.1155/2022/6425133 (PMC9167133; doi:10.1155/2022/6425133)
Supplement: Supplementary Materials — Figure S1: comparison of CEA, CA19-9, and CA72-4 between the MSS group and the MSI group. Statistical analysis was performed by the Wilcoxon rank-sum test. ∗p < 0.05. Figure S2: comparison of CEA, CA19-9, and CA72-4 between the HER2 negative group and the HER2 positive group. Table S1: comparison of 18 heavy metals between healthy controls and GC patients. Table S2: comparison of 18 heavy metals between the MSS group and the MSI group. Table S3: comparison of CEA, CA19-9, and CA72-4 between the MSS group and the MSI group. Table S4: comparison of 18 heavy metals between the HER2 negative group and the HER2 positive group. Table S5: comparison of CEA, CA19-9, and CA72-4 between the HER2 negative group and the HER2 positive group. Table S6: correlations analysis among MSI, HER2 gene amplification, and 18 heavy metals. Table S7: correlations analysis among MSI, HER2 gene amplification, 3 biomarkers, and 18 heavy metals. [file 6425133.f1.zip › 6425133.f1/Table S2.docx]

| Table S2: Comparison of 18 heavy metals between the MSS group and the MSI group. | | | |
| --- | --- | --- | --- |
|  | MSI (n=18) | MSS (n=87) |  |
| Heavy metals | Median+IQR | Median+IQR | *p* value |
| V | 0.29 (0.17-0.78) | 0.26 (0.15-0.49) | 0.75 |
| Cr | 2.33 (1.66-3.01) | 2.47 (2.02-3.02) | 0.65 |
| Mn | 12.47 (9.67-16.04) | 11.1 (9.24-13.69) | 0.28 |
| Co | 0.37 (0.30-0.54） | 0.22 (0.1-0.46) | 0.13 |
| Ni | 0.89（0.52-1.19) | 0.77 (0.37-1.4) | 0.83 |
| Cu | 927 (826.4-1042) | 882.3 (755.4-1010) | 0.18 |
| Zn | 5.65 (4.97-6.09) | 5.68 (4.88-6.37) | 0.92 |
| Ga | 0.26 (0.01-0.46) | 0.01 (0.01-0.19) | 0.019 |
| As | 0.93 (0-1.35) | 0.77 (0.24-1.45) | 0.87 |
| Se | 165.8 (107.8-198.3) | 141.1 (111.1-195) | 0.54 |
| Sr | 20.66 (18.22-30.69) | 23.44 (18.37-29.38) | 0.68 |
| Cd | 0.68 (0.22-1.44) | 0.59 (0.16-1.48) | 0.72 |
| Sn | 0 (0-0.01) | 0.01 (0-0.01) | 0.32 |
| Sb | 0.01 (0-0.21) | 0.01 (0-0.01) | 0.15 |
| Ba | 44.73 (32.56-79.07) | 45.33 (32.67-66.36) | 0.69 |
| Hg | 0 (0-0.01) | 0.01 (0-0.01) | 0.4 |
| Tl | 0 (0-0.01) | 0 (0-0.01) | 0.84 |
| Pb | 12 (9.56-15.4) | 11.65 (7.55-14.51) | 0.37 |
| MSI: Microsatellite instability; MSS: microsatellite-stable; IQR: interquartile range. | | | |

**p*<0.05 was considered significant.
