# Supplementary material for: The Influence of Heavy Metals on Gastric Tumorigenesis
Source: J Oncol. 2022 May 28;2022:6425133. doi: 10.1155/2022/6425133 (PMC9167133; doi:10.1155/2022/6425133)
Supplement: Supplementary Materials — Figure S1: comparison of CEA, CA19-9, and CA72-4 between the MSS group and the MSI group. Statistical analysis was performed by the Wilcoxon rank-sum test. ∗p < 0.05. Figure S2: comparison of CEA, CA19-9, and CA72-4 between the HER2 negative group and the HER2 positive group. Table S1: comparison of 18 heavy metals between healthy controls and GC patients. Table S2: comparison of 18 heavy metals between the MSS group and the MSI group. Table S3: comparison of CEA, CA19-9, and CA72-4 between the MSS group and the MSI group. Table S4: comparison of 18 heavy metals between the HER2 negative group and the HER2 positive group. Table S5: comparison of CEA, CA19-9, and CA72-4 between the HER2 negative group and the HER2 positive group. Table S6: correlations analysis among MSI, HER2 gene amplification, and 18 heavy metals. Table S7: correlations analysis among MSI, HER2 gene amplification, 3 biomarkers, and 18 heavy metals. [file 6425133.f1.zip › 6425133.f1/Table S3.docx]

| Table S3: Comparison of CEA, CA19-9 and CA72-4 between the MSS group and the MSI group. | | | |
| --- | --- | --- | --- |
|  | MSI (n=10) | MSS (n=38) |  |
| Biomarkers | Median+IQR | Median+IQR | *p* value |
| CEA | 2.39 (1.25-8.33) | 2.83 (1.49-7.26) | 0.56 |
| CA19-9 | 9.92 (6.85-17.98) | 10.78 (6.66-31.91) | 0.93 |
| CA72-4 | 4.03 (2.07-19.54) | 1.64 (1.05-4.94) | 0.025 |
| CA19-9: carbohydrate antigen 19-9; CA72-4: carbohydrate antigen 72-4 ;CEA: carcinoembryonic antigen; MSI: Microsatellite instability; MSS: microsatellite-stable; IQR: interquartile range. | | | |

**p*<0.05 was considered significant.
