# Supplementary material for: The Influence of Heavy Metals on Gastric Tumorigenesis
Source: J Oncol. 2022 May 28;2022:6425133. doi: 10.1155/2022/6425133 (PMC9167133; doi:10.1155/2022/6425133)
Supplement: Supplementary Materials — Figure S1: comparison of CEA, CA19-9, and CA72-4 between the MSS group and the MSI group. Statistical analysis was performed by the Wilcoxon rank-sum test. ∗p < 0.05. Figure S2: comparison of CEA, CA19-9, and CA72-4 between the HER2 negative group and the HER2 positive group. Table S1: comparison of 18 heavy metals between healthy controls and GC patients. Table S2: comparison of 18 heavy metals between the MSS group and the MSI group. Table S3: comparison of CEA, CA19-9, and CA72-4 between the MSS group and the MSI group. Table S4: comparison of 18 heavy metals between the HER2 negative group and the HER2 positive group. Table S5: comparison of CEA, CA19-9, and CA72-4 between the HER2 negative group and the HER2 positive group. Table S6: correlations analysis among MSI, HER2 gene amplification, and 18 heavy metals. Table S7: correlations analysis among MSI, HER2 gene amplification, 3 biomarkers, and 18 heavy metals. [file 6425133.f1.zip › 6425133.f1/Table S4.docx]

| Table S4: Comparison of 18 heavy metals between the HER2 negative group and the HER2 positive group. | | | |
| --- | --- | --- | --- |
|  | HER2 Negative (n=69) | HER2 Positive (n=28) |  |
| Heavy metals | Median+IQR | Median+IQR | *p* value |
| V | 0.26 (0.17-0.56) | 0.29 (0.18-0.49) | 0.86 |
| Cr | 2.55 (1.95-3.15) | 2.44 (2.07-3.01) | 0.84 |
| Mn | 11.26 (8.85-13.87) | 11.67 (9.52-13.32) | 0.9 |
| Co | 0.33 (0.13-0.52) | 0.21 (0.10-0.44) | 0.26 |
| Ni | 0.89 (0.44-1.46) | 0.69 (0.33-1.29) | 0.33 |
| Cu | 912.3 (768-1029) | 857.3 (709.8-981.8) | 0.064 |
| Zn | 5.68 (4.96-6.5) | 5.69 (4.86-6.25) | 0.95 |
| Ga | 0.04 (0-0.27) | 0.02 (0.01-0.23) | 0.68 |
| As | 0.76 (0.03-1.385) | 0.93 (0.61-1.63) | 0.24 |
| Se | 145.6 (113.4-200.8) | 137 (95.25-191.9) | 0.27 |
| Sr | 23.55 (18.73-29.57) | 21.9 (17.34-27.89) | 0.26 |
| Cd | 0.62 (0.19-1.52) | 0.45 (0.07-1.40) | 0.4 |
| Sn | 0 (0-0.01) | 0.01 (0-0.01) | 0.034 |
| Sb | 0.01 (0-0.06) | 0.01 (0-0.1) | 0.28 |
| Ba | 46.39 (35.74-66.08) | 53.61 (31.88-69.36) | 0.9 |
| Hg | 0 (0-0.01) | 0.01 (0-0.01) | 0.002 |
| Tl | 0 (0-0.01) | 0.01 (0-0.05) | 0.015 |
| Pb | 11.42 (7.4-14.27) | 12.16 (9.03-14.36) | 0.37 |
| HER2: human epidermal growth factor receptor type 2; IQR: interquartile range. | | | |

**p*<0.05 was considered significant.
