# Supplementary material for: The Influence of Heavy Metals on Gastric Tumorigenesis
Source: J Oncol. 2022 May 28;2022:6425133. doi: 10.1155/2022/6425133 (PMC9167133; doi:10.1155/2022/6425133)
Supplement: Supplementary Materials — Figure S1: comparison of CEA, CA19-9, and CA72-4 between the MSS group and the MSI group. Statistical analysis was performed by the Wilcoxon rank-sum test. ∗p < 0.05. Figure S2: comparison of CEA, CA19-9, and CA72-4 between the HER2 negative group and the HER2 positive group. Table S1: comparison of 18 heavy metals between healthy controls and GC patients. Table S2: comparison of 18 heavy metals between the MSS group and the MSI group. Table S3: comparison of CEA, CA19-9, and CA72-4 between the MSS group and the MSI group. Table S4: comparison of 18 heavy metals between the HER2 negative group and the HER2 positive group. Table S5: comparison of CEA, CA19-9, and CA72-4 between the HER2 negative group and the HER2 positive group. Table S6: correlations analysis among MSI, HER2 gene amplification, and 18 heavy metals. Table S7: correlations analysis among MSI, HER2 gene amplification, 3 biomarkers, and 18 heavy metals. [file 6425133.f1.zip › 6425133.f1/Table S5.docx]

| Table S5: Comparison of CEA, CA19-9 and CA72-4 between the HER2 negative group and the HER2 positive group. | | | |
| --- | --- | --- | --- |
|  | Her2 negative (n=31) | Her2 positive (n=13) |  |
| Biomarkers | Median+IQR | Median+IQR | *p* value |
| CEA | 2.79 (1.43-9.09) | 3.31 (1.52-6.13) | 0.8 |
| CA19-9 | 10.65 (7.04-18.44) | 8.95 (6.19-53.49) | 0.67 |
| CA72-4 | 1.67 (1.15-4.89) | 2.85 (1.51-7.96) | 0.13 |
| CA19-9: carbohydrate antigen 19-9; CA72-4: carbohydrate antigen 72-4; CEA: carcinoembryonic antigen; HER2: human epidermal growth factor receptor type 2; IQR: interquartile range. | | | |
