# Supplementary material for: The Influence of Heavy Metals on Gastric Tumorigenesis
Source: J Oncol. 2022 May 28;2022:6425133. doi: 10.1155/2022/6425133 (PMC9167133; doi:10.1155/2022/6425133)
Supplement: Supplementary Materials — Figure S1: comparison of CEA, CA19-9, and CA72-4 between the MSS group and the MSI group. Statistical analysis was performed by the Wilcoxon rank-sum test. ∗p < 0.05. Figure S2: comparison of CEA, CA19-9, and CA72-4 between the HER2 negative group and the HER2 positive group. Table S1: comparison of 18 heavy metals between healthy controls and GC patients. Table S2: comparison of 18 heavy metals between the MSS group and the MSI group. Table S3: comparison of CEA, CA19-9, and CA72-4 between the MSS group and the MSI group. Table S4: comparison of 18 heavy metals between the HER2 negative group and the HER2 positive group. Table S5: comparison of CEA, CA19-9, and CA72-4 between the HER2 negative group and the HER2 positive group. Table S6: correlations analysis among MSI, HER2 gene amplification, and 18 heavy metals. Table S7: correlations analysis among MSI, HER2 gene amplification, 3 biomarkers, and 18 heavy metals. [file 6425133.f1.zip › 6425133.f1/Table S7.docx]

| Table S7: Correlations analysis among MSI, HER2 gene amplification, 3 biomarkers and 18 heavy metals. | | | | | | | | | | | | | | | | | | | | | | | | |
| --- | --- | --- | --- | --- | --- | --- | --- | --- | --- | --- | --- | --- | --- | --- | --- | --- | --- | --- | --- | --- | --- | --- | --- | --- |
|  | | MSI | HER2 | CEA | CA19-9 | CA72-4 | V | Cr | Mn | Co | Ni | Cu | Zn | Ga | As | Se | Sr | Cd | Sn | Sb | Ba | Hg | Tl | Pb |
| MSI | r |  | -0.11 | 0.13 | 0.06 | 0.06 | 0.27 | -0.09 | 0.10 | 0.05 | -0.12 | 0.00 | -0.16 | 0.14 | -0.09 | -0.05 | 0.13 | -0.07 | -0.05 | 0.29 | 0.21 | -0.03 | -0.07 | -0.06 |
|  | *p* |  | 0.46 | 0.39 | 0.68 | 0.68 | 0.07 | 0.54 | 0.53 | 0.74 | 0.46 | 1.00 | 0.29 | 0.38 | 0.56 | 0.73 | 0.40 | 0.64 | 0.76 | 0.06 | 0.17 | 0.83 | 0.67 | 0.72 |
| HER2 | r | -0.11 |  | -0.14 | 0.07 | 0.20 | -0.12 | 0.18 | -0.13 | -0.27 | 0.21 | -0.27 | 0.09 | 0.12 | -0.06 | 0.10 | -0.14 | 0.15 | 0.28 | -0.12 | -0.04 | -0.14 | 0.04 | 0.05 |
|  | *p* | 0.46 |  | 0.35 | 0.63 | 0.19 | 0.45 | 0.23 | 0.39 | 0.08 | 0.17 | 0.08 | 0.57 | 0.45 | 0.69 | 0.54 | 0.36 | 0.33 | 0.06 | 0.43 | 0.77 | 0.36 | 0.80 | 0.77 |
| CEA | r | 0.13 | -0.14 |  | -0.04 | 0.17 | -0.07 | -0.01 | -0.14 | -0.11 | -0.04 | -0.22 | -0.06 | 0.00 | -0.10 | -0.05 | 0.06 | -0.16 | -0.07 | -0.07 | 0.02 | -0.06 | 0.19 | -0.04 |
|  | *p* | 0.39 | 0.35 |  | 0.82 | 0.26 | 0.67 | 0.94 | 0.36 | 0.49 | 0.80 | 0.15 | 0.71 | 0.99 | 0.53 | 0.74 | 0.71 | 0.30 | 0.67 | 0.65 | 0.87 | 0.70 | 0.23 | 0.82 |
| CA19-9 | r | 0.06 | 0.07 | -0.04 |  | -0.04 | -0.07 | -0.05 | 0.01 | 0.17 | 0.07 | 0.16 | 0.19 | -0.01 | -0.08 | -0.07 | -0.12 | 0.02 | 0.02 | 0.07 | -0.02 | 0.51^***^ | -0.01 | -0.09 |
|  | *p* | 0.68 | 0.63 | 0.82 |  | 0.79 | 0.67 | 0.75 | 0.96 | 0.27 | 0.65 | 0.29 | 0.21 | 0.95 | 0.60 | 0.67 | 0.42 | 0.91 | 0.89 | 0.66 | 0.88 | 0.00 | 0.94 | 0.54 |
| CA72-4 | r | 0.06 | 0.20 | 0.17 | -0.04 |  | -0.05 | 0.71^***^ | 0.05 | -0.05 | 0.74^***^ | 0.16 | 0.24 | 0.04 | -0.04 | -0.06 | -0.06 | -0.07 | -0.03 | -0.06 | -0.02 | 0.01 | 0.05 | 0.01 |
|  | *p* | 0.68 | 0.19 | 0.26 | 0.79 |  | 0.77 | 0.00 | 0.75 | 0.75 | 0.00 | 0.29 | 0.12 | 0.80 | 0.80 | 0.68 | 0.70 | 0.66 | 0.85 | 0.70 | 0.92 | 0.95 | 0.77 | 0.94 |
| V | r | 0.27 | -0.12 | -0.07 | -0.07 | -0.05 |  | 0.19 | -0.08 | 0.02 | 0.00 | -0.15 | -0.02 | -0.03 | -0.08 | 0.00 | 0.06 | -0.11 | -0.03 | -0.04 | -0.12 | -0.04 | -0.08 | -0.04 |
|  | *p* | 0.07 | 0.45 | 0.67 | 0.67 | 0.77 |  | 0.23 | 0.59 | 0.90 | 0.99 | 0.34 | 0.90 | 0.85 | 0.60 | 0.98 | 0.69 | 0.47 | 0.83 | 0.82 | 0.43 | 0.80 | 0.63 | 0.78 |
| Cr | r | -0.09 | 0.18 | -0.01 | -0.05 | 0.71^***^ | 0.19 |  | 0.00 | -0.18 | 0.61^***^ | 0.11 | 0.20 | -0.17 | -0.21 | -0.05 | -0.05 | 0.20 | -0.05 | -0.16 | -0.11 | -0.06 | -0.06 | 0.06 |
|  | *p* | 0.54 | 0.23 | 0.94 | 0.75 | 0.00 | 0.23 |  | 1.00 | 0.26 | 0.00 | 0.46 | 0.19 | 0.26 | 0.17 | 0.76 | 0.75 | 0.20 | 0.73 | 0.31 | 0.47 | 0.69 | 0.68 | 0.71 |
| Mn | r | 0.10 | -0.13 | -0.14 | 0.01 | 0.05 | -0.08 | 0.00 |  | -0.19 | -0.16 | 0.4^**^ | 0.36^*^ | -0.12 | 0.34^*^ | 0.05 | 0.08 | 0.17 | -0.04 | -0.08 | 0.03 | 0.24 | 0.28 | -0.05 |
|  | *p* | 0.53 | 0.39 | 0.36 | 0.96 | 0.75 | 0.59 | 1.00 |  | 0.21 | 0.31 | 0.01 | 0.02 | 0.44 | 0.03 | 0.75 | 0.61 | 0.27 | 0.78 | 0.59 | 0.82 | 0.11 | 0.06 | 0.76 |
| Co | r | 0.05 | -0.27 | -0.11 | 0.17 | -0.05 | 0.02 | -0.18 | -0.19 |  | 0.21 | 0.36^*^ | -0.07 | 0.09 | -0.14 | -0.10 | 0.10 | -0.05 | -0.08 | -0.02 | 0.10 | -0.03 | -0.31* | 0.20 |
|  | *p* | 0.74 | 0.08 | 0.49 | 0.27 | 0.75 | 0.90 | 0.26 | 0.21 |  | 0.16 | 0.02 | 0.67 | 0.55 | 0.38 | 0.53 | 0.53 | 0.73 | 0.63 | 0.88 | 0.52 | 0.86 | 0.04 | 0.18 |
| Ni | r | -0.12 | 0.21 | -0.04 | 0.07 | 0.74^***^ | 0.00 | 0.61^***^ | -0.16 | 0.21 |  | 0.23 | 0.22 | -0.17 | -0.05 | -0.16 | -0.02 | 0.15 | 0.02 | -0.02 | 0.03 | 0.02 | -0.07 | 0.06 |
|  | *p* | 0.46 | 0.17 | 0.80 | 0.65 | 0.00 | 0.99 | 0.00 | 0.31 | 0.16 |  | 0.13 | 0.16 | 0.28 | 0.73 | 0.31 | 0.92 | 0.34 | 0.91 | 0.89 | 0.85 | 0.90 | 0.64 | 0.71 |
| Cu | r | 0.00 | -0.27 | -0.22 | 0.16 | 0.16 | -0.15 | 0.11 | 0.4^**^ | 0.36^*^ | 0.23 |  | 0.36^*^ | 0.06 | 0.09 | -0.09 | 0.13 | 0.18 | -0.05 | 0.08 | 0.16 | 0.27 | -0.03 | -0.01 |
|  | *p* | 1.00 | 0.08 | 0.15 | 0.29 | 0.29 | 0.34 | 0.46 | 0.01 | 0.02 | 0.13 |  | 0.02 | 0.68 | 0.57 | 0.57 | 0.41 | 0.24 | 0.75 | 0.59 | 0.31 | 0.08 | 0.82 | 0.94 |
| Zn | r | -0.16 | 0.09 | -0.06 | 0.19 | 0.24 | -0.02 | 0.20 | 0.36^*^ | -0.07 | 0.22 | 0.36^*^ |  | 0.08 | 0.13 | 0.15 | -0.32* | 0.24 | -0.21 | 0.05 | -0.11 | 0.39^**^ | 0.06 | 0.09 |
|  | *p* | 0.29 | 0.57 | 0.71 | 0.21 | 0.12 | 0.90 | 0.19 | 0.02 | 0.67 | 0.16 | 0.02 |  | 0.59 | 0.42 | 0.33 | 0.03 | 0.12 | 0.16 | 0.77 | 0.48 | 0.01 | 0.69 | 0.55 |
| Ga | r | 0.14 | 0.12 | 0.00 | -0.01 | 0.04 | -0.03 | -0.17 | -0.12 | 0.09 | -0.17 | 0.06 | 0.08 |  | 0.16 | 0.00 | 0.03 | -0.11 | -0.06 | -0.06 | 0.42^**^ | 0.00 | -0.13 | 0.07 |
|  | *p* | 0.38 | 0.45 | 0.99 | 0.95 | 0.80 | 0.85 | 0.26 | 0.44 | 0.55 | 0.28 | 0.68 | 0.59 |  | 0.30 | 0.98 | 0.84 | 0.47 | 0.70 | 0.68 | 0.00 | 0.97 | 0.40 | 0.63 |
| As | r | -0.09 | -0.06 | -0.10 | -0.08 | -0.04 | -0.08 | -0.21 | 0.34^*^ | -0.14 | -0.05 | 0.09 | 0.13 | 0.16 |  | 0.03 | 0.32^*^ | -0.04 | 0.02 | 0.14 | 0.37^*^ | 0.05 | 0.28 | -0.08 |
|  | *p* | 0.56 | 0.69 | 0.53 | 0.60 | 0.80 | 0.60 | 0.17 | 0.03 | 0.38 | 0.73 | 0.57 | 0.42 | 0.30 |  | 0.82 | 0.03 | 0.80 | 0.88 | 0.35 | 0.01 | 0.77 | 0.07 | 0.59 |
| Se | r | -0.05 | 0.10 | -0.05 | -0.07 | -0.06 | 0.00 | -0.05 | 0.05 | -0.10 | -0.16 | -0.09 | 0.15 | 0.00 | 0.03 |  | -0.04 | 0.08 | -0.06 | -0.07 | 0.12 | 0.11 | -0.11 | 0.47^**^ |
|  | *p* | 0.73 | 0.54 | 0.74 | 0.67 | 0.68 | 0.98 | 0.76 | 0.75 | 0.53 | 0.31 | 0.57 | 0.33 | 0.98 | 0.82 |  | 0.81 | 0.60 | 0.71 | 0.67 | 0.44 | 0.46 | 0.47 | 0.00 |
| Sr | r | 0.13 | -0.14 | 0.06 | -0.12 | -0.06 | 0.06 | -0.05 | 0.08 | 0.10 | -0.02 | 0.13 | -0.32* | 0.03 | 0.32^*^ | -0.04 |  | 0.18 | -0.01 | -0.17 | 0.46^**^ | -0.06 | 0.06 | -0.06 |
|  | *p* | 0.40 | 0.36 | 0.71 | 0.42 | 0.70 | 0.69 | 0.75 | 0.61 | 0.53 | 0.92 | 0.41 | 0.03 | 0.84 | 0.03 | 0.81 |  | 0.24 | 0.95 | 0.26 | 0.00 | 0.68 | 0.70 | 0.68 |
| Cd | r | -0.07 | 0.15 | -0.16 | 0.02 | -0.07 | -0.11 | 0.20 | 0.17 | -0.05 | 0.15 | 0.18 | 0.24 | -0.11 | -0.04 | 0.08 | 0.18 |  | 0.29 | -0.14 | 0.09 | 0.01 | 0.12 | 0.4^**^ |
|  | *p* | 0.64 | 0.33 | 0.30 | 0.91 | 0.66 | 0.47 | 0.20 | 0.27 | 0.73 | 0.34 | 0.24 | 0.12 | 0.47 | 0.80 | 0.60 | 0.24 |  | 0.05 | 0.38 | 0.55 | 0.92 | 0.43 | 0.01 |
| Sn | r | -0.05 | 0.28 | -0.07 | 0.02 | -0.03 | -0.03 | -0.05 | -0.04 | -0.08 | 0.02 | -0.05 | -0.21 | -0.06 | 0.02 | -0.06 | -0.01 | 0.29 |  | -0.04 | -0.10 | -0.04 | -0.05 | 0.00 |
|  | *p* | 0.76 | 0.06 | 0.67 | 0.89 | 0.85 | 0.83 | 0.73 | 0.78 | 0.63 | 0.91 | 0.75 | 0.16 | 0.70 | 0.88 | 0.71 | 0.95 | 0.05 |  | 0.79 | 0.53 | 0.79 | 0.74 | 1.00 |
| Sb | r | 0.29 | -0.12 | -0.07 | 0.07 | -0.06 | -0.04 | -0.16 | -0.08 | -0.02 | -0.02 | 0.08 | 0.05 | -0.06 | 0.14 | -0.07 | -0.17 | -0.14 | -0.04 |  | -0.21 | 0.18 | -0.06 | -0.02 |
|  | *p* | 0.06 | 0.43 | 0.65 | 0.66 | 0.70 | 0.82 | 0.31 | 0.59 | 0.88 | 0.89 | 0.59 | 0.77 | 0.68 | 0.35 | 0.67 | 0.26 | 0.38 | 0.79 |  | 0.16 | 0.23 | 0.69 | 0.87 |
| Ba | r | 0.21 | -0.04 | 0.02 | -0.02 | -0.02 | -0.12 | -0.11 | 0.03 | 0.10 | 0.03 | 0.16 | -0.11 | 0.42^**^ | 0.37^*^ | 0.12 | 0.46^**^ | 0.09 | -0.10 | -0.21 |  | -0.23 | 0.03 | 0.07 |
|  | *p* | 0.17 | 0.77 | 0.87 | 0.88 | 0.92 | 0.43 | 0.47 | 0.82 | 0.52 | 0.85 | 0.31 | 0.48 | 0.00 | 0.01 | 0.44 | 0.00 | 0.55 | 0.53 | 0.16 |  | 0.14 | 0.87 | 0.63 |
| Hg | r | -0.03 | -0.14 | -0.06 | 0.51^***^ | 0.01 | -0.04 | -0.06 | 0.24 | -0.03 | 0.02 | 0.27 | 0.39^**^ | 0.00 | 0.05 | 0.11 | -0.06 | 0.01 | -0.04 | 0.18 | -0.23 |  | -0.09 | -0.08 |
|  | *p* | 0.83 | 0.36 | 0.70 | 0.00 | 0.95 | 0.80 | 0.69 | 0.11 | 0.86 | 0.90 | 0.08 | 0.01 | 0.97 | 0.77 | 0.46 | 0.68 | 0.92 | 0.79 | 0.23 | 0.14 |  | 0.57 | 0.59 |
| Tl | r | -0.07 | 0.04 | 0.19 | -0.01 | 0.05 | -0.08 | -0.06 | 0.28 | -0.31* | -0.07 | -0.03 | 0.06 | -0.13 | 0.28 | -0.11 | 0.06 | 0.12 | -0.05 | -0.06 | 0.03 | -0.09 |  | -0.06 |
|  | *p* | 0.67 | 0.80 | 0.23 | 0.94 | 0.77 | 0.63 | 0.68 | 0.06 | 0.04 | 0.64 | 0.82 | 0.69 | 0.40 | 0.07 | 0.47 | 0.70 | 0.43 | 0.74 | 0.69 | 0.87 | 0.57 |  | 0.70 |
| Pb | r | -0.06 | 0.05 | -0.04 | -0.09 | 0.01 | -0.04 | 0.06 | -0.05 | 0.20 | 0.06 | -0.01 | 0.09 | 0.07 | -0.08 | 0.47^**^ | -0.06 | 0.4^**^ | 0.00 | -0.02 | 0.07 | -0.08 | -0.06 |  |
|  | *p* | 0.72 | 0.77 | 0.82 | 0.54 | 0.94 | 0.78 | 0.71 | 0.76 | 0.18 | 0.71 | 0.94 | 0.55 | 0.63 | 0.59 | 0.00 | 0.68 | 0.01 | 1.00 | 0.87 | 0.63 | 0.59 | 0.70 |  |

MSI: Microsatellite instability; HER2: human epidermal growth factor receptor type 2. **p*<0.05 was considered significant.
