# Supplementary figures and images for: The Influence of Heavy Metals on Gastric Tumorigenesis
Source: J Oncol. 2022 May 28;2022:6425133. doi: 10.1155/2022/6425133 (PMC9167133; doi:10.1155/2022/6425133)

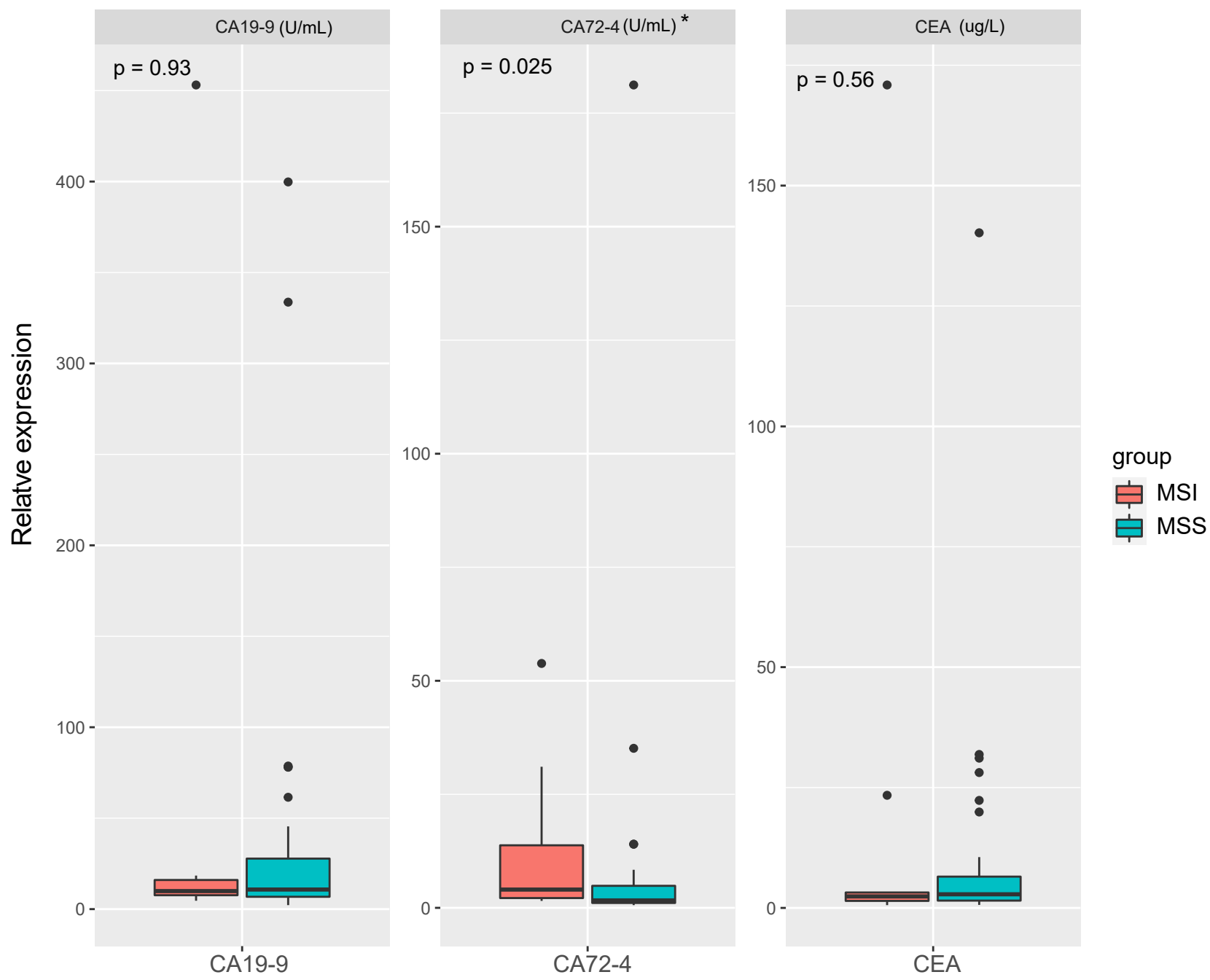

Supplement: Supplementary Materials — Figure S1: comparison of CEA, CA19-9, and CA72-4 between the MSS group and the MSI group. Statistical analysis was performed by the Wilcoxon rank-sum test. ∗p < 0.05. Figure S2: comparison of CEA, CA19-9, and CA72-4 between the HER2 negative group and the HER2 positive group. Table S1: comparison of 18 heavy metals between healthy controls and GC patients. Table S2: comparison of 18 heavy metals between the MSS group and the MSI group. Table S3: comparison of CEA, CA19-9, and CA72-4 between the MSS group and the MSI group. Table S4: comparison of 18 heavy metals between the HER2 negative group and the HER2 positive group. Table S5: comparison of CEA, CA19-9, and CA72-4 between the HER2 negative group and the HER2 positive group. Table S6: correlations analysis among MSI, HER2 gene amplification, and 18 heavy metals. Table S7: correlations analysis among MSI, HER2 gene amplification, 3 biomarkers, and 18 heavy metals. [file 6425133.f1.zip › 6425133.f1/Figure S1.pdf]
